# Host predisposition by endogenous Transforming Growth Factor-β1 overexpression promotes pulmonary fibrosis following bleomycin injury

**DOI:** 10.1186/1476-9255-4-18

**Published:** 2007-09-20

**Authors:** Yussef Haider, Andrea P Malizia, Dominic T Keating, Mary Birch, Annette Tomlinson, Gail Martin, Mark WJ Ferguson, Peter P Doran, Jim J Egan

**Affiliations:** 1 School of Biological Sciences, University of Manchester, Manchester, UK; 2 National Heart and Lung Transplant Program, Mater Misericordiae University Hospital, University College Dublin, Dublin; 3 Genome Resource Unit, Dublin Molecular Medicine Centre, Mater Misericordiae University Hospital, University College Dublin, Dublin, Ireland; 4 Advanced Lung Disease Programme, Mater Misericordiae University Hospital, University College Dublin, 44 Eccles Street, Dublin 7, Ireland

## Abstract

**Background:**

Idiopathic Pulmonary Fibrosis (IPF) is a progressive diffuse disease involving the lung parenchyma. Despite recent advances, the molecular mechanisms of the initiation and progression of this disease remain elusive. Previous studies have demonstrated TGFβ1 as a key effector cytokine in the development of lung fibrosis.

**Methods:**

In this study we have used a transgenic mouse based strategy to identify the effect of overexpression of this key effector mediator on the development of pulmonary fibrosis in response to exogenous injury. We bred two lines (line 25 and 18) of transgenic mice (Tr+) that overexpressed active TGFβ1. Three-month old transgenic and wild type mice were subsequently wounded with intraperitoneal bleomycin. Mice were sacrificed at 6 weeks post-bleomycin and their lungs analysed histologically and biochemically.

**Results:**

The severity of lung fibrosis was significantly greater in the Tr+ mice compared to the wild type mice. Using an oligonucleotide microarray based strategy we identified discrete patterns of gene expression contributing to TGFβ1 associated pulmonary fibrosis.

**Conclusion:**

This data emphasises the importance of a host predisposition in the form of endogenous TGFβ1, in the development of pulmonary fibrosis in response to an exogenous injury.

## Background

Idiopathic pulmonary fibrosis (IPF) is a progressive diffuse fibrotic process involving the lung parenchyma. It is a chronic, progressively debilitating and ultimately fatal disorder [1]. Treatment options are limited and lung transplantation may be offered to a minority of patients.

Recent studies have demonstrated that the outcome from disease was determined by the burden of collections of activated fibroblasts, fibroblastic foci, and not the extent of histological inflammation [2-5].

In IPF, exogenous mediators are thought to precipitate the lung injury, and in synergy with genetic factors contribute to the disease. These genetic factors are increasingly thought to play an important role, with familial patterning of IPF already described. Efforts to identify genetic loci linked to this disease have thus far been inconclusive [6]. Extensive experimental evidence has identified, TGFβ1 as a central regulator of tissue fibrosis at multiple sites. Evidence from studies of fibrotic disorders, including renal and liver fibrosis, supports that TGFβ1 may play a novel role in fibrogenesis by promoting epithelial-mesenchymal transition (EMT) and activating fibroblasts to myofibroblasts [7-9]. In mature epithelial cells, TGFβ1 can initiate EMT through activation of intracellular signalling molecules [10,11]. EMT contributes to the degeneration of epithelial structures and to the generation of fibroblasts in chronic fibrotic disorders [8,12,13]. While targeted overproduction of TGF-β1 is associated with an increase in pulmonary fibrosis, antagonising its effects prevent the fibrotic process [14]. Reviewing potential candidate pathways that might offer novel therapeutic targets to treat IPF, Antoniou KM reported antibodies to TGFβ1 significantly reduced the cytokine experimental lung and kidney fibrosis and a receptor antagonist to this cytokine decreased accumulation of lung collagen induced by bleomycin [15]. Several approaches to reduce TGFβ1 levels have also been evaluated in human tissue *in vivo*, showing downregulation of the fibrotic process by IFN-1b, which may occur both directly and indirectly by modification of the fibroblast response to reduced TGFβ1. [16,17].

In this study we evaluated TGF-β1 gene overexpression in isolation and the impact of an exogenous injury in the setting of a host genetically predisposed by endogenous TGF-β1 gene overexpression. Furthermore we sought to characterise the molecular mechanisms underpinning the development of the resultant fibrosis utilising gene array techniques.

## Methods

### TGFβ1 transgenic mice

The mice were originally engineered by microinjection of a DNA fragment into the nuclei of one-cell mouse embryos. The DNA fragment containing an altered porcine TGFβ1 cDNA associated with an albumin promoter to ensure the preferential expression of the active form of TGFβ1 from the liver, with resultant high circulating levels [18]. Mouse embryos were obtained from mating of F1 hybrid mice (C57BL6 × CBA background). Two lines of mice (line 18 and line 25) were purchased (Nancy Sanderson, National Institute Of Health, Bethesda, MD, USA).

The transgene was expressed in both sexes of the line 18 mice. The mice were bred by crossing a positive with a wild type animal. In the line 25 mice, only the male mice expressed the transgene. Therefore, the mice were bred by setting up a harem consisting of a positive male animal and three F1 females. Mice were housed under pathogen-free conditions and husbanded according to Home Office regulations.

On day 0 mice were given intraperitoneal bleomycin (BLM) or phosphate buffer solution (PBS) in three divided doses (0.5 ml volume) over a course of 5 days. They were observed on a daily basis and sacrificed on day 42. Mice were divided into 6 groups (n = 8/group).

### Characterisation of TGFβ1 Tr+ transgenic mice

Mouse-tail snips, measuring approximately 0.25 cm, were incubated with proteinase K overnight at 55°C and DNA was extracted the following day using Phenol/Chloroform/Isoamylalcohol method followed by washing-step in 70% ethanol.

TGFβ1 quantification was performed using a PAI-1/Luciferase assay (PAIL). PAIL assay is a quantitative bioassay based upon active TGFβ's ability to stimulate the expression of Plasminogen Activator Inhibitor 1 (PAI-1) [19]. The assay uses mink lung epithelial cells (MLEC's) (a kind gift from Dr Dan Rifkin, New York University Medical Center, New York), which have been stably transfected with a gene for Luciferase activity and its expression is regulated and promoted by a truncated PAI-1 promoter construct. TGFβ1 therefore regulates Luciferase activity via PAI-1 promoter. Luciferase activity in MLEC cell lysates was measured in a luminometer.

### Histological analysis

Liver and lung tissue sections were stained with haematoxylin and eosin and Masson's trichrome, which determines collagen deposition and localization. Lung fibrosis was graded histologically by an established scoring system [20].

Immunohistochemical analysis was performed as previously described [21]. In brief, paraffin sections were stained with rabbit anti-reticulin (Sigma, UK), rabbit anti-TGFβ1 (Santa Cruz, CA, USA) and its receptors (TGFβ1R1, TGFβ1R2, TGFβ1R3) (Santa Cruz, CA, USA) (1:100). Antibody binding was visualized using a biotinylated secondary antibody, avidine conjugated peroxidase (ABC method; Vector Laboratories) and 3,3' diaminobenzidine tetrachloride (DAB) as a substrate and hematoxylin as counterstain.

### Collagen assay

For collagen determination we employed a hydroxyproline assay technique. Briefly after death, lungs were removed and weighed. 6 M hydrochloric acid was added to each sample, then sealed and placed in an oven overnight at 110°C. Excess acid was removed by evaporation and hydrolyzed samples were dissolved in 1 ml of PBS. The samples were aliquoted adding Chloramine T reagent equally to each sample. After 20 minutes of mixing, 1 ml of p-DAB reagent (p-dimethyl-amino-benzaldehyde) was added and the mixture incubated at 60°C. The colour signals were measured in a spectrophotometer at 550 nm, and compared to a standard curve.

### Microarray analysis

RNA isolation, cDNA synthesis, *in vitro *transcription and microarray analysis were performed as previously reported [22]. Arrays were scanned with a confocal scanner (Affymetrix). All *in vitro *time points were microarrayed in duplicate.

Image files were obtained through Affymetrix GeneChip software (MAS5). Subsequently robust multichip analysis (RMA) was performed [23,24]. Expression data was further probed to identify those genes whose expression is altered [25]. Expression data following injury was compared to control and a signal log ratio of 0.6 or greater (equivalent to a fold change in expression of 1.5 or greater) was taken to identify significant differential regulation. Using normalised RMA values, Unsupervised Average Linkage Hierarchical Cluster Analysis was performed [26]. Functional annotation of differentially expressed genes was curated via the publicly available Onto-Compare and Gene-Ontology (GO) databases [27].

## Results and discussion

### TGF-β1 transgenic mice develop severe liver fibrosis

**Figure 1:**
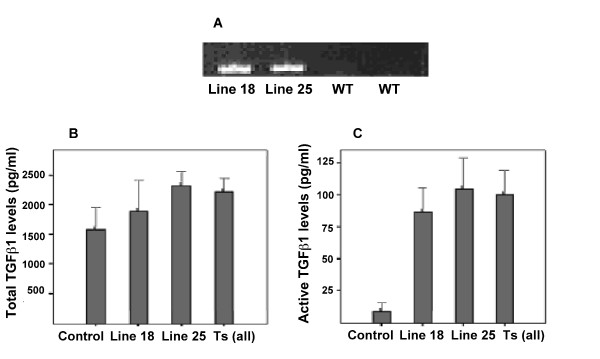
**Characterisation of TGFβ1 transgenic mice**. A. Expression of the transgene in wild type and both line 18 and line 25 transgenic mice was assessed by PCR using TGFβ1 sequence specific primers. This figure is a representative agarose gel post amplification indicating expression of the TGFβ1 transgene in both line 18 and line 25 Tr+ transgenic mice (*Lanes 1 and 2). Lanes 3 and 4 *show absence of transgene in wild type mice. Figure B and C show PAIL lumineriferase assay results. To determine the effect of the transgene on circulating TGFβ1, both total (B) and active (C) TGFβ1 concentrations in sera was determined.

Following breeding, TGFβ1 expression was confirmed by PCR amplification in Tr+ TGFβ1 transgenic mice (Figure 1a). To determine the effect of the TGFβ1 transgene in these mice, serum levels of both total and active TGFβ1 were determined. The Tr+ transgenic mice had higher levels of total TGFβ1 (2.2 ng/ml, SEM 0.23) compared to Tr- wild types (1.58 ng/ml, SEM 0.39), though this comparison did not reach statistical significance (p = 0.16) (Figure 1b); while, Tr+ transgenic mice had higher plasma levels of active TGFβ1 (mean 98.1 pg/ml, SEM 16.1) compared to Tr- wild types (mean 9.37, SEM 6.6) (p < 0.01). Individually, the line 18 mice had a similar level of active TGFβ1 (mean 87 pg/ml, SEM 19.2) to the line 25 mice (105.5 pg/ml, SEM 24.5) (Figure 1c).

Having demonstrated altered DNA and protein expression in TGFβ1-transgenic mice we sought to determine the effect of TGFβ1 overexpression on tissue phenotype.

**Figure 2:**
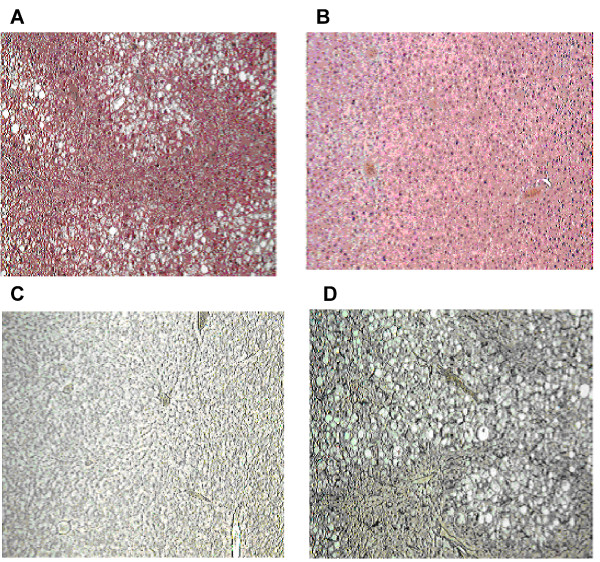
**TGFβ1 overexpression induces severe liver fibrosis**. A. Shown are representative micrographs following haematoxylin/eosin staining indicating severe liver fibrosis in TGFβ1 transgene expressing mice. B shows normal Tr- wild type mice liver tissue stained by haematoxylin/eosin. C. The deposition of the pro-collagen, reticulin, was also determined using specific monoclonal antibody anty-reticulin by immunohistochemistry in Tr- WT mice liver tissue sections. D. Whilst low abundance staining is seen in wild type liver, expression of reticulin is dramatically enhanced in the Tr+ TGFβ1 transgenic mice.

Tr+ transgenic mouse livers were histologically abnormal as early as 1 month, though the most marked changes were seen from 3 months onwards. This consisted of extensive cellular degeneration, vacuolisation, fibrosis and architectural disruption (Figure 2a), compared to Tr- wild type mouse liver (Figure 2b). Staining for the pre-collagen, reticulin signalling was higher in transgenic mice tissue than wild type, confirming the presence of ongoing tissue fibrosis (Figure 2c–d). Tissue changes were most pronounced in the line 25 mice, but also present in line 18 mice, while wild type mice had normal liver architecture and normal reticulin levels.

These data demonstrate overexpression of TGFβ1 in Tr+ transgenic mice and detail the alterations in phenotype, providing a model for the assessment of the contribution of this important effector cytokine to the fibrotic milieu *in vivo*.

Overexpression of TGFβ1 in the liver leads to a severe liver fibrosis. Fibrotic liver phenotype presented at 1 month with the injury being most severe from 3 months onwards. Of note was the finding of enhanced reticulin deposition in the fibrotic tissue versus Tr- wild type mice. These data provide evidence that overexpression of TGFβ1 in mouse liver promotes de novo fibrosis, even in the absence of other pro-fibrotic stimuli.

### Overexpression of TGFβ1 does not cause de novo lung fibrosis

**Figure 3:**
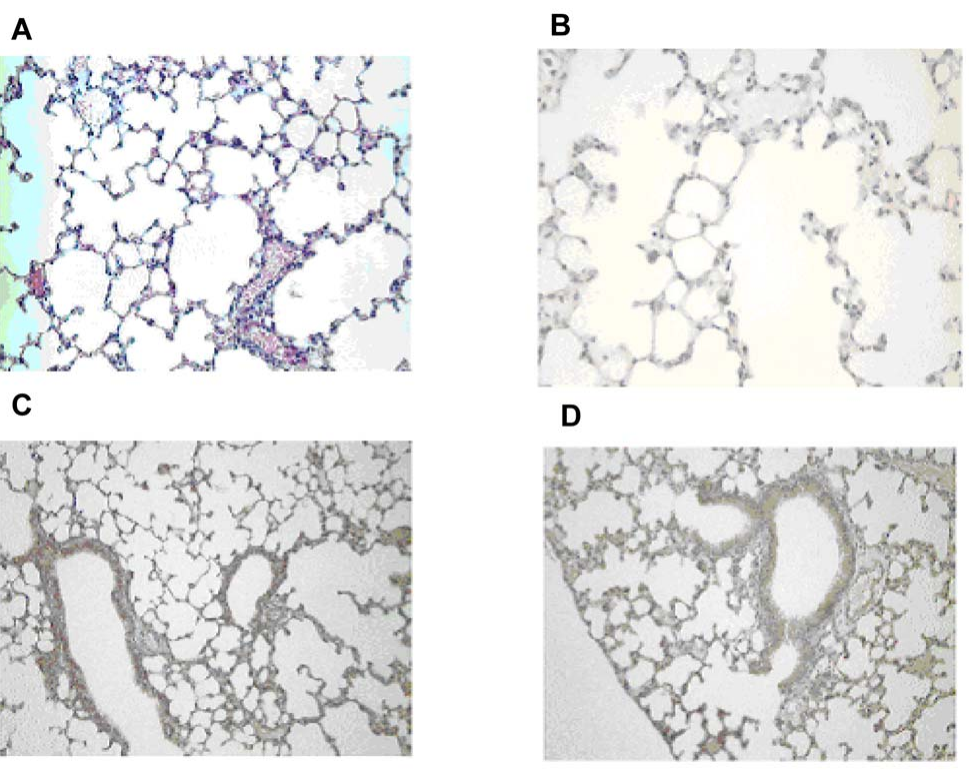
**Absence of lung fibrosis in TGFβ1 overexpressing mice**. Figure A shows haematoxylin and eosin staining of Tr+ TGFβ1 transgenic lung tissue. Of note was the absence of fibrosis in transgenic mice. To determine the molecular events underpinning this process we determined the expression of TGFβ1 (B), Type I receptor for TGFβ1 (C) and the TGFβ1 Type II receptor (D) by immunohistochemistry. As can be seen TGFβ1 and its receptors are present in abundance in lung tissue from these mice, indicating a normal TGFβ1 signalling cascade in Tr+ pulmonary tissue.

Having demonstrated the molecular effect of TGFβ1 overexpression and its effect on mouse liver we examined the impact of Tr+ TGFβ1 transgenic expression on mouse lung. Of note was the finding that transgenic mouse lungs (Figure 3a) showed no evidence of *de novo *fibrosis at any time point studied.

To determine the putative mechanism underpinning this tissue specific finding we characterised the expression of TGFβ1 in transgenic mouse lung. Figure 3b shows staining for active TGFβ1 in lung tissue, providing evidence that whilst TGFβ1 is present in the lung it does not produce a fibrotic response. Having determined the presence of active TGFβ1 in these lungs, expression of TGFβ1 receptors was investigated. Of note was the finding that, Tr+ transgenic mouse lung was found to contain an abundance of both Type I (Figure 3c) and type II (Figure 3d) TGFβ1 receptors.

These data demonstrate that *de novo *tissue fibrosis in response to TGFβ1 overexpression is tissue specific. In the setting of the lung, active TGFβ1 does not produce a fibrotic phenotype despite an abundance of both type I and type II receptors. The data presented herein lend weight to the hypothesis that TGFβ1 contributes to lung fibrosis *in vivo *through the interplay with other factors, and is not sufficient, in itself to drive lung fibrosis.

### Oligonucleotide microarray analysis identifies distinct patterns of gene expression underpinning lung injury

The data generated in the histological studies identified that TGFβ1 overexpression is insufficient to establish pulmonary fibrosis. However we determined the effect of a second insult with bleomycin on TGFβ1- and WT-transgenic mice.

**Figure 4:**
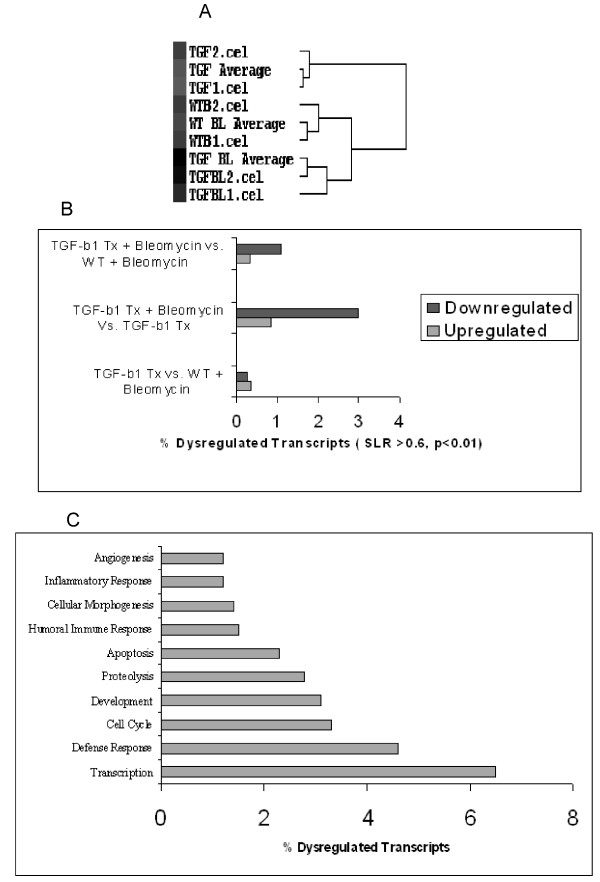
**Oligonucleotide microarray analysis reveals coordinate patterns of gene expression in response to bleomycin lung injury**. A. Gene expression in Bleomycin treated Tr- Wild Type (WT BL, WTB1, WTB2) and Tr+ TGFβ1 trasgenic mice (TGF BL, TGFBL1, TGFBL2), and untreated Tr+ TGFβ1 trasgenic mice (TGF, TGF1, TGF2) was assessed using Affymetrix Mouse Genome 430_2 oligonucleotide microarrays in duplicate (data are reported in the cluster dendogram as single analysis and average: cel1, cel2 and average, respectively). Average and actual expression values for all significantly dysregulated genes were used as input in unsupervised hierarchical cluster visualization. Shown is a representative cluster dendrograms indicating separation of the conditions based on gene expression profiles, highlighting an high homology (based on the t-score) of both bleomycin treated group, respect to untreated Tr+ transgenic mice group. Figure B summarises the total number of genes found to be significantly altered in each comparison (Tr+ and bleomycin vs Tr- WT and bleomycin; Tr+ and bleomycin vs Tr+; Tr+ vs Tr- WT and bleomycin). A high number of altered genes were found to be upregulated and dowwnregulated in bleomycin treated Tr+ vs Tr+ group. C. To further annotate the pulmonary fibrosis associated transcriptome, significantly perturbed genes from bleomycin treated Tr+ vs Tr+ group were used as input in searches of the Gene Ontology database to identify the biological function of the altered genes.

To determine the molecular events subserving the TGFβ1 mediated exacerbation of lung fibrosis we utilised an oligonucleotide microarray based strategy to identify altered key transcripts. Specifically, we probed the molecular contribution to the repetitive injury, namely the expression changes induced by TGFβ1 overexpression and the expression changes that result from bleomycin exposure in these Tr+ transgenic mice. Affymetrix Mouse Genome 430_2 microarrays were used to determine gene expression levels in lung tissue from a) untreated Tr+ TGFβ1 transgenic mice b) Tr- wild type mice treated with bleomycin and c) bleomycin treated Tr+ TGFβ1 transgenic mice, to identify the overall pattern of gene expression in this experiment. Significant changes in gene expression were associated with these tissue cohorts (-0.6 < SLR > 0.6, and p < 0.05) (Figure 4a). Distinct patterns of coordinate gene expression were observed throughout the exposures, with substantial transcriptomic effects in terms of both up and downregulation of gene expression separating the sample groups.

Of the 45,101 gene sequences represented on the Affymetrix Mouse Genome 430_2 oligonucleotide microarray, 6.2% (2812 genes) were found to be significantly altered in all three groups. Exposure of Tr+ TGFβ1 transgenic mice to bleomycin elicited a major gene expression response with a total of 3.9% significantly altered transcripts (1724 genes) in compare to untreated Tr+ TGFβ1 transgenic mice. To probe molecular basis of TGFβ1 exacerbation of lung injury the expression profiles of both bleomycin-treated WT and TGFβ1 transgenic mice were compared, showing 640 significant gene expression changes between these groups (1.4% of the transcripts represented on the microarray) (Figure 4b).

To further annotate the transcriptomic differences between the study groups' ontological classification of molecular function was investigated by using Gene Ontology database (Figure 4c). By using Gene Ontology database, Bleomycin TR+ vs TR+ altered transcripts were classified and grouped in functional families in correlation with their significant role in fibrosis development. Altered fibrosis-associated genes, which drive angiogenesis, inflammatory response, immune response and apoptosis, in response to bleomycin in TR+ mice were found dysregulated, as also previously reported [28-30]. In addition, we found a large number of significantly altered genes that function in the regulation of cellular morphogenesis, development and gene transcription. Bleomycin-exposed TR+ TGFβ1 transgenic mice show overall altered gene expression profile which correlate with cellular morphogenesis and gene transcription, enhanced cellular functions which trigger fibrosis development.

Tables 1 and 2 highlight the genes whose mRNA levels were most strikingly altered in bleomycin injured and Tr+ TGFβ1 transgenic mice.

**Table 1:** Genes undergoing most significant upregulation in Bleomycin-exposed Tr+ TGFβ1 transgenic mice in compare to Tr+ untreated mice.

**Accession No**.	**Gene name**	**SLRs**
M12573.1	heat shock protein, 70 kDa 1	0.586101
BB746075	dipeptidyl peptidase 7	0.586311
X67128.1	rearranged T-cell receptor beta chain	0.586724
AF061744.1	FYN binding protein	0.588032
NM_010724.1	proteosome (prosome, macropain) subunit, beta type 8	0.590169
L42293.1	O-acyltransferase 1 (Soat1)	0.591774
NM_008979.1	protein tyrosine phosphatase, non-receptor type 8	0.592489
L78253.1	killer cell lectin-like receptor, subfamily A, member 8	0.597584
BB206460	phosphatidylinositol membrane-associated	0.599792
NM_009099.1	tripartite motif protein 30	0.600294
NM_007655.1	immunoglobulin-associated alpha (Iga)	0.600366
U29539.1	retinoic acid-inducible E3 protein	0.602024
BF301241	immunoglobulin kappa chain variable region	0.602451
M34563.1	CD28 antigen (Cd28)	0.602497
AW322280	keratin complex 2, basic, gene 8	0.60367
NM_009049.1	endocrine-specific protein 18	0.604619
BF301241	immunoglobulin kappa chain variable region	0.606074
NM_011487.1	signal transducer and activator of transcription 4	0.606996
BC002043.1	cyclin-dependent kinase inhibitor 1A	0.608647
M33266.1	small inducible cytokine B subfamily (Cys-X-Cys)	0.61223
AW227993	complement component 1, q subcomponent, beta polypeptide	0.612379
L05631.1	IL2-inducible T-cell kinase (Itk)	0.612786
NM_009952.1	cAMP response element binding protein (Creb1)	0.613665
AF274046.1	nuclear protein 95 (Np95)	0.615783
NM_010234.1	FBJ osteosarcoma oncogene	0.617241
NM_008328.1	interferon activated gene 203	0.617767
NM_011580.1	thrombospondin 1	0.621648
M26071.1	coagulation factor III	0.622067
AV075715	Clusterin	0.623468
BM124741	heat shock protein 25 kDa 2	0.625063

**Table 2:** Genes undergoing most significant downregulation in Bleomycin-exposed Tr+ TGFβ1 transgenic mice in compare to Tr+ untreated mice.

**Accession No**.	**Gene name**	**SLRs**
NM_033525.1	nephronectin	-3.104277
NM_008508.1	loricrin	-2.9579485
NM_008218.1	hemoglobin alpha, adult chain 1	-2.5698385
AF071431.1	beta globin	-2.4558515
NM_009868.1	cadherin 5	-2.383406
NM_009502.1	vinculin	-2.2180055
AB015595.1	calcitonin receptor-like receptor precursor	-2.217319
BB623587	integrin alpha8	-2.155636
NM_007925.1	elastin	-2.1294445
M34962.1	histocompatibility 2, L region	-2.0658385
AW550625	procollagen, type III, alpha 1	-2.056252
X14480.1	nidogen 1	-2.027124
AK013851.1	G protein gamma 3 linked gene	-1.928728
BG060909	stearoyl-Coenzyme A desaturase 2	-1.922733
BC004850.1	twisted gastrulation protein	-1.854459
BE573195	epithelial membrane protein 2	-1.848788
AI324124	synuclein, alpha	-1.80537
NM_008475.1	keratin complex 2, basic, gene 4	-1.7794
AB041350.1	type IV collagen alpha 5 chain	-1.756065
BM211336	ferrochelatase	-1.745825
AK013376.1	amyloid beta (A4) precursor-like	-1.725242
NM_011594.1	tissue inhibitor of metalloproteinase 2	-1.705896
AF252873.1	CXC chemokine MIP-2gamma precursor	-1.7039455
AY075134.1	T-box 4	-1.7018585
NM_009100.1	repetin	-1.683903
NM_021099.2	kit oncogene	-1.642024
BM239368	tumor differentially expressed 1	-1.6415555
U08020.1	collagen pro-alpha-1 type I chain m	-1.6209215
AF128892.1	protein kinase Piccolo	-1.615523
AF017989.1	secreted frizzled-related seq. protein 2	-1.5890315

Table 1 indicates a large number of altered genes, which are largely recognized as mediators of immunological function. Further annotation of these upregulated genes identified a large number of genes involved in cytokine signalling. Further, the transcripts whose expression was found to be altered in response to bleomycin exposure included a large number of extracellular matrix and matrix regulation associated genes, key effectors molecules in the development of tissue fibrosis.

### TGFβ1 overexpression primes mouse lung for fibrotic injury following bleomycin exposure

Having demonstrated that overexpression of TGFβ1, whilst initiating severe fibrosis in mouse liver, does not cause *de novo *lung fibrosis; we determined the effect of escalating doses of bleomycin on TGFβ1 and WT transgenic mice.

Exposure to 1500 IU of bleomycin resulted in 5 mice (Tr+ line 25) developing lung fibrosis compared to only 2 in the Tr- wild type group. 4500 IU of bleomycin showed fibrosis in all 8 mice of the Tr+ line 25, compared to 6 mice in the Tr+ line 18 group and 5 mice in the Tr- wild type group. These data determine the dose response nature of lung injury following exposure to bleomycin.

**Figure 5:**
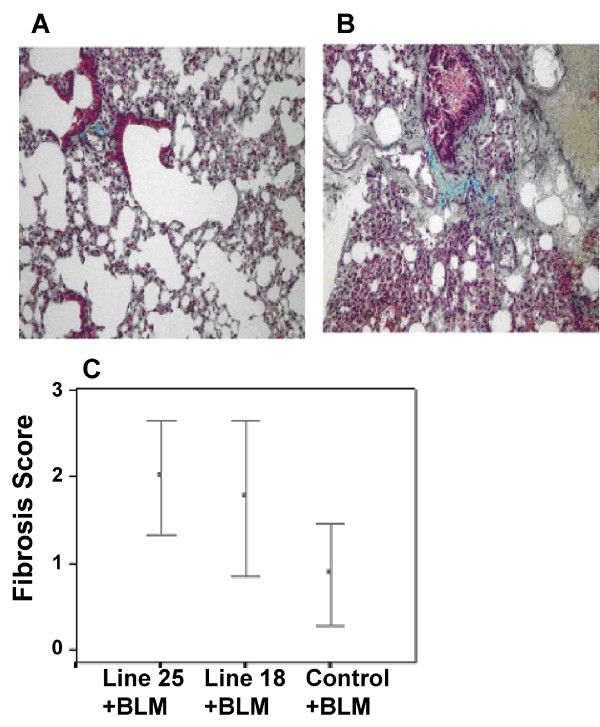
**TGFβ1 overexpression induces pronounced fibrotic response following bleomycin exposure**. Tissue fibrosis was assessed in both Tr- wild type (A) and Tr+ transgenic (B) mice lung following exposure to 4500 IU bleomycin, as previously described. Shown are representative micrographs following haematoxylin/eosin staining of lung tissue, demonstrating fibrotic response in bleomycin treated wild type mouse lung that is significantly more severe in tissue from Tr+ TGFβ1 transgenic mice, suggesting that overexpression of the TGFβ1 transgene exacerbates subsequent lung injury. C. To quantify this fibrotic effect, fibrosis scores were determined as described. The graph shows enhanced fibrosis scores in Tr+ TGFβ1 transgenic mice versus their Tr- wild type counterparts in response to bleomycin exposure.

Lung fibrosis induced by 4500 IU bleomycin in the Tr- wild type group was a mild patchy lung injury (Figure 5a). Tr+ transgenic mice, following exposure to comparable and smaller doses of bleomycin demonstrated marked lung injury hallmarked by grossly thickened alveolar walls, inflammation, fibroblast proliferation and collagen deposition in a peribronchial, interstitial and sub pleural distribution (Figure 5b).

To validate these tissue observations the fibrosis score in bleomycin treated mice was determined as described. The Tr+ transgenic-bleomycin group had greater fibrosis scores (mean 1.88, SEM 0.27) than the Tr- wild type-bleomycin group (mean 0.875, SEM 0.295) (p < 0.05). Tr+ line 25-bleomycin group had the highest mean score (mean 2.0, SEM 0.32) (p < 0.05) (Figure 5c) while the Tr+ line 18-bleomycin group also had a score of 1.75 (SEM 0.45). The PBS vehicle Tr- wild type group had scores of 0 (n = 8). These data further demonstrate the exacerbation of bleomycin elicited lung injury in mice overexpressing TGFβ1.

**Figure 6:**
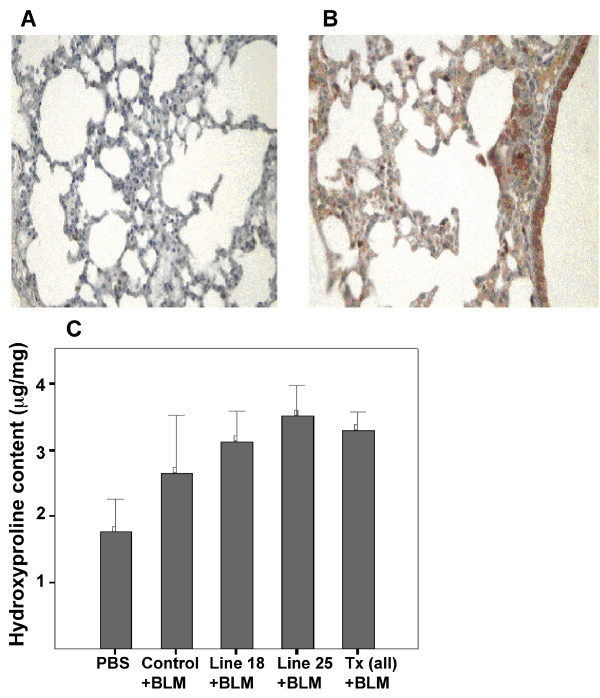
**Enhanced TGFβ1 immunostaining and collagen production in bleomycin treated Tr+ TGFβ1 transgenic mice**. A, B. To determine the role of TGFβ1 in the induction of lung fibrosis in mice treated with bleomycin, immunostaining for active TGFβ1 was performed as previously described. Shown are representative micrographs following immunostaining for TGFβ1 in the lung of bleomycin treated, Tr- wild type (A) and Tr+ TGFβ1 transgenic (B) mice. Expression of TGFβ1 is present in both tissue specimens but is substantially enhanced in the Tr+ TGFβ1 transgenic mice. C. Total collagen was determined by hydroxyproline assay as previously described. Collagen production was significantly enhanced in all bleomycin-wounded mice versus control PBS exposed, and in particular collagen deposition has been found higher in Tr+ TGFβ1 transgenic mice than in Tr- Wild Type ones. BLM, Bleomycin.

Finally, we determined the tissue distribution of TGFβ1 in lung tissue from both Tr- wild type and Tr+ transgenic mice following exposure to bleomycin. Immunostaining for TGFβ1 demonstrated marked expression of TGFβ1 in both wild type (Figure 6a) and Tr+ transgenic (Figure 6b) mice following bleomycin exposure. Of note is the particularly strong expression in TGFβ1 transgenic mice, suggesting that bleomycin exposure elicits a more pronounced TGFβ1 response in Tr+ transgenic versus wild type mouse lung.

### Bleomycin induced pulmonary fibrosis in Tr+ mice enhances collagen deposition

Having determined the fibrotic response induced by exposure to 4500 IU of bleomycin in both wild type and TGFβ1 transgenic mouse lung tissue we further investigated collagen production in lung tissue following exposure to bleomycin.

*De novo *collagen production was assessed using hydroxyproline assay. The lung hydroxyproline content was higher in the Tr+ transgenic-bleomycin group (mean 3.3 μg/mg, SEM 0.11) than in the Tr- wild type-bleomycin group (mean 2.4 μg/mg, SEM 0.33)(p < 0.05) or the unwounded PBS group (mean 1.76 μg/mg, SEM 0.16) (Figure 6).

In summary, the fibrotic response in Tr+ transgenic mice is dominated firstly by immune mediators reacting to bleomycin exposure and causing lung injury and secondly by genes (TGFβ1) contributing to the deposition of extracellular matrix. These data lend further weight to the hypothesis that pulmonary fibrosis is a result of combined injury from both endogenous and exogenous mediators and provides important evidence for the interplay of these factors in the development of tissue fibrosis. Further analysis of these transcriptomic alterations will reveal the exact mechanism of the synergistic lung injury induced by TGFβ1 overexpression and bleomycin injury.

## Conclusion

In this study we have utilized transgenic mice to simulate TGFβ1-related genetic predisposition to external stimuli, rather than a tissue specific TGFβ1 transgenic model. We have used a combination of gene overexpression and exposure to an exogenous agent to further define the complex nature of the initiation and progression of pulmonary fibrosis. In common with most other complex disorders, this data suggests that one single factor is insufficient to promote pulmonary fibrosis in isolation.

Increasing evidence has shown that it is the interplay of myriad biological factors that promote the development of this disease. TGFβ1 has been explored in depth in the context of IPF due to its well-described pro-fibrotic injury. In this study overexpression of the gene encoding TGFβ1 in mice was shown to initiate severe liver fibrosis as evidenced by histology. However, of note was the finding that overexpression of the gene did not result in a *de novo *fibrotic response in mouse lung. To probe the mechanism at work in the lung we determined and showed that the key components of TGFβ1 signalling were present in the lung, despite the lack of fibrotic responses. This data emphasises that a genetic predisposition in isolation is insufficient to promote pulmonary fibrosis.

Despite several limitations [31], bleomycin-induced fibrosis in an animal model is the most common method for studying fibrosis. Some bleomycin-based model in mice replicate human pathologic features of IPF, including fibroproliferation within the lung parenchyma, and hence pathologic mechanisms discerned in the mouse are worthy of consideration, specifically it is recognized as a source of epithelial cell injury which is considered central to IPF in the human setting. The importance of TGFβ1 to the development of lung fibrosis, was however, demonstrated by the lung response to bleomycin. As compared with their wild type treated counterparts, the transgenic mice showed a much more severe fibrosis, as evidenced by both histological analysis and deposition of collagen in the lung. These data raise the possibility that overexpression of the TGFβ1 gene, in the germline, results in a subsequent inappropriate response to bleomycin exposure. This finding may explain a number of key features of the disease including the susceptibility of some patients to more severe progression and the multifactorial nature of the pathology. Defects in the TGFβ1 gene resulting in increased TGFβ1 production, or locally induced TGFβ1 increases may only be sufficient to cause a fibrotic injury when acting in concert with other factors, thus reinforcing the hypothesis that IPF development is due to the interplay of a number of factors.

We defined the fibrotic response in the transgenic animal population following epithelial cell injury, and characterised the transcriptomic changes. The data showed distinct patterns of gene expression driving the tissue response to injury.

Chambers *et al*. have profiled human foetal lung fibroblast global gene expression in response to TGFβ1 revealing the induction of Inhibitor of Differentiation-1 (ID1) and providing evidence of smooth muscle cell phenotypic switching [32]. Also, Liu *et al*. analyzed the lung gene expression in bleomycin-exposed rats to identify genes that may be involved in fibrosis, and identified FIZZ1 as critical mediator in myofibroblast differentiation [33].

Our study demonstrated coordinate expression of distinct gene families with the response dominated by mediators of the immune response, suggesting the importance of immunological events in the initiation of lung injury. However, repetitive lung injury, that is the synergistic activity of bleomycin and TGFβ1 led to a switch in this transcriptomic response. The double injury mice were characterised by molecular patterns that are hallmarks of fibrosis, including extracellular matrix and cell growth and regulation gene clusters. These data provide evidence for the mechanism underpinning the exacerbated fibrosis seen in transgenic mice at the tissue level. TGFβ1 overexpression primes the cellular machinery to produce a fibrotic transcriptome when exposed to bleomycin emphasising further the importance of this cytokine in the development of tissue fibrosis. Although TGFβ1 is a critical initial key response factor after injury, it must be recognised that in humans other cytokines including CTGF, collagen and angiogenic mediators are involved in the pathogenesis of pulmonary fibrosis.

Genes that encode for mediators of the immune response (biological processes, defence response, humoral immune response and inflammatory response) were found to be altered in significant numbers in the setting of the fibrotic lung. Exposure to this exogenous profibrogenic agent elicits an immune response characterised by upregulation of immune pathway genes [34]. Furthermore the development of tissue fibrosis has previously been demonstrated to be a result of, at least in part, an imbalance in the Th1/Th2 cytokine milieu, where the Th2 pro-fibrotic response is dominant [35].

Of note was the finding of a relatively large number of development-associated genes whose expression was significantly altered. The inappropriate recapitulation of developmental processes has been shown to be a disease-initiating event in the setting of renal fibrosis [36-38]. The finding of these perturbed developmental networks in the lung may indicate a similar role of development gone awry in the setting of lung fibrosis.

These data demonstrate enhanced collagen deposition in TGFβ1 transgenic mice compared to wild type mouse in response to bleomycin wounding. This increase in collagen production, coupled with the observed enhanced active TGFβ1 immunostaining in transgenic mice provide further evidence for the pathogenomic role of TGFβ1 in the initiation and progression of lung fibrosis.

The data contained herein provide further evidence for the complex interplay of numerous factors in the development of IPF and provide evidence for the synergistic activity of genetic and exogenous mediators in eliciting fibrosis in the lung. Further investigations will focus on characterising the combination of these factors in patients with IPF, specifically with attention paid to the role of the TGFβ1 gene as a prime for subsequent lung injury.

## Abbreviations

IPF- Idiopathic Pulmonary Fibrosis;

TGFβ1- Transforming Growth Factor β1.

## Authors' contributions

YH completed the animal work and drafted the manuscript. APM completed the functional analysis and wrote the manuscript. DTK completed the microarray and functional analysis. MB, AT, and GM completed the animal work. MWJF oversaw the animal work and wrote the manuscript. PPD oversaw the microarray analysis. JJE conceived, coordinated, funded the project and wrote the manuscript.

All authors read and approved the final manuscript.
